# A synthesis of ecological and evolutionary determinants of bat diversity across spatial scales

**DOI:** 10.1186/s12898-018-0174-z

**Published:** 2018-06-11

**Authors:** Franciele Parreira Peixoto, Pedro Henrique Pereira Braga, Poliana Mendes

**Affiliations:** 1 Programa de Pós-Graduação em Ecologia e Evolução, Instituto de Ciências Biológicas, Departamento de Ecologia, Universidade Federal de Goiás, Goiânia, Goiás 74001-970 Brazil; 2 Graduate Program in Biology, Department of Biology, Concordia University, Loyola Campus, 7141 Sherbrooke Street West, Montréal, QC H2R 2K7 Canada; 3 Laboratório de Ecologia e Conservação da Biodiversidade, Pós-Graduação em Ecologia de Ecossistemas, Universidade Vila Velha, Centro Biopráticas, Rua Mercúrio, Boa Vista I, Vila Velha, Espírito Santo 29101-420 Brazil

**Keywords:** Chiroptera, Communities, Scale hierarchy, Guild, Diversity gradients, Evolutionary history, Spatial scales

## Abstract

**Background:**

Diversity patterns result from ecological to evolutionary processes operating at different spatial and temporal scales. Species trait variation determine the spatial scales at which organisms perceive the environment. Despite this knowledge, the coupling of all these factors to understand how diversity is structured is still deficient. Here, we review the role of ecological and evolutionary processes operating across different hierarchically spatial scales to shape diversity patterns of bats—the second largest mammal order and the only mammals with real flight capability.

**Main body:**

We observed that flight development and its provision of increased dispersal ability influenced the diversification, life history, geographic distribution, and local interspecific interactions of bats, differently across multiple spatial scales. Niche packing combined with different flight, foraging and echolocation strategies and differential use of air space allowed the coexistence among bats as well as for an increased diversity supported by the environment. Considering distinct bat species distributions across space due to their functional characteristics, we assert that understanding such characteristics in Chiroptera improves the knowledge on ecological processes at different scales. We also point two main knowledge gaps that limit progress on the knowledge on scale-dependence of ecological and evolutionary processes in bats: a geographical bias, showing that research on bats is mainly done in the New World; and the lack of studies addressing the mesoscale (i.e. landscape and metacommunity scales).

**Conclusions:**

We propose that it is essential to couple spatial scales and different zoogeographical regions along with their functional traits, to address bat diversity patterns and understand how they are distributed across the environment. Understanding how bats perceive space is a complex task: all bats can fly, but their perception of space varies with their biological traits.

**Electronic supplementary material:**

The online version of this article (10.1186/s12898-018-0174-z) contains supplementary material, which is available to authorized users.

## Background

Describing diversity patterns and determining their structuring processes are critical tasks in ecology. Since the relative importance of processes delineating diversity varies across spatial scales [1–3], it is essential to define the spatial scale at which patterns have been described to produce generalizations [4, 5]. By disregarding the space-dependence of diversity determinants, one is likely to fail in finding consistent results and may mistakenly associate processes to probable incorrect patterns [6]. Over the past three decades, it has been argued that regional and local diversity are hierarchically related [2, 7–9]. For instance, species diversity results from a balance between ecological and historical-evolutionary processes simultaneously acting in a hierarchy of scales [1, 3, 10, 11]. Historical and evolutionary processes, operating at larger scales, determine diversity through speciation and biota exchange [1, 11]. Evolutionary processes not only generate diversity, but also shape and constrain phenotypes, and thus, species abilities to integrate ecological interactions [11]. Therefore, processes acting at larger scales ultimately influence local diversity and other processes acting at the local scale [1]. Conversely, local diversity is mainly structured by ecological processes, which may limit species diversity through, for example, negative interspecific interactions (e.g., predation and competition), and environmental filtering.

Ecologists often define assemblages (i.e., taxonomically delimited communities; Fauth et al. [12]) for practical reasons. Proper scale delimitation to capture interspecific interactions depends on the studied taxon and the scientific question proposed. Ant communities will certainly respond to the environment at a different extension than bat communities due to their differences in perception of the environment (e.g., Lessard et al. [13], Villalobos and Arita [14]). Moreover, species occurring in sympatry do not necessarily directly interact, since they may belong to different ensembles (i.e., taxonomically and functionally delimited assemblages; Fauth et al. [12]) and might differently use the space (e.g., Kalko [15]). Hence, since the scale at which an organism interacts is dependent on its life history characteristics [16, 17], the balance between evolutionary and ecological processes determining diversity will not be the same for distinct taxonomic groups.

Here, we review the current knowledge on the role of ecological and evolutionary processes acting across different scales to shape bat distribution and diversity patterns (see the Additional file 1: Methods used in this review). We start by discussing evolutionary processes determining bat diversification, and consecutively following a spatially hierarchical framework, from broader to more local spatial scales. Although we acknowledge that many processes act simultaneously across multiple scales, we delimited our discussion based on the main processes linked to each hierarchical level. We do not aim to explore and define the boundaries between each scale; whereas we focused on the evolutionary and ecological processes per se that structure bat distribution and diversity at each spatial scale. We focus our study on bats (Chiroptera), the second largest mammal order, with nearly 1270 described species [18]. Bats are the only mammals with real flight capability. They are distributed across all continents, except the poles, and are more diverse in the tropics [19, 20]. Almost all bats are exclusively nocturnal [21]. Also, bats feed on a wide variety of resources (e.g., arthropods, vertebrates, fruit, pollen and blood), play important ecological roles and provide economic benefits, such as pollination, seed dispersion and pest control [18, 22].

### Global scale: historical, phylogenetic and geographical components

We consider the global scale of bat diversity patterns across three main axes: *historical*, which includes the life-history of bats, including the development of key innovations that allowed bats to occupy the globe; *phylogenetic*, which englobes the diversification patterns across the different clades; and, *geographical*, which explores the resulting patterns of bat diversity across large spatial scales.

#### Evolutionary history of bats, key innovations and adaptive radiation

**Fig. 1:**
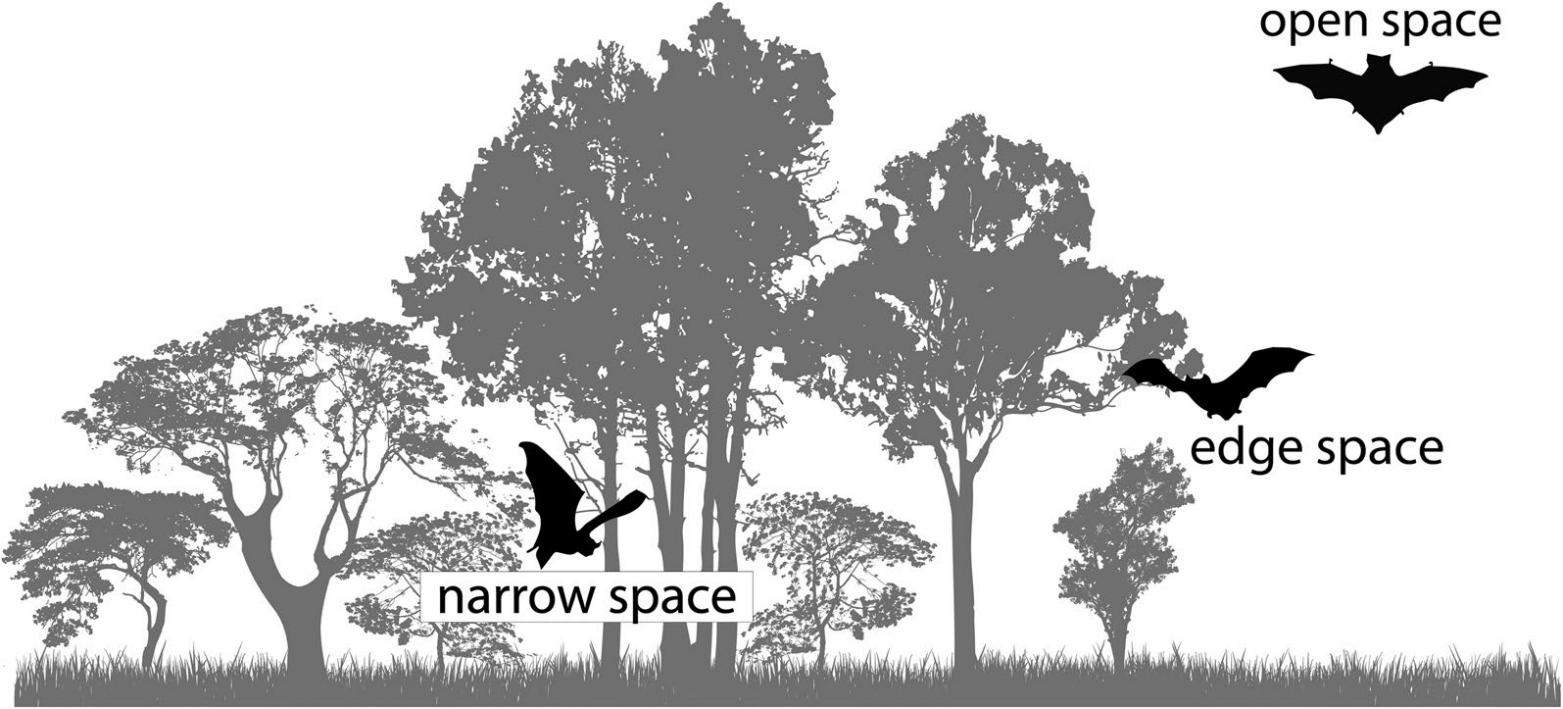
Air-space used by bats. Open-space species fly higher and further from ground obstacles; edge-space species fly across the vegetation edge and clearings; and, narrow-space species fly across the vegetation (Adapted from Kalko et al. [102])

The order Chiroptera was usually divided into two suborders: (1) Megachiroptera (fruit bats), composed by a single family from the Old World (Pteropodidae, with about 170 species); and (2) Microchiroptera [23], which holds other 17 families distributed across all continents (but see Eick et al. [24] and Jones and Teeling [25]) (Fig. 1). Microchiropteran bats use laryngeal echolocation (a form of sonar) to guide themselves and to locate prey. Old World fruit bats (Pteropodidae) lack laryngeal echolocation and high-frequency hearing [26, 27]. A different subdivision of Chiroptera has been proposed based on molecular data: Yangochiroptera and Yinpterochiroptera [28, 29]. The principal difference between this subdivision and the previous is the placement of the families Craseonycteridae, Hipposideridae, Megadermatidae, Rhinolophidae, and Rhinopomatidae (all echolocating taxa) with Pteropodidae (a non-echolocating taxon) to form the group Yinpterochiroptera. This last subdivision has an important impact in the knowledge on evolution of echolocation in bats, since it implies that echolocation was lost in Pteropodidae or that it developed multiple times across the evolutionary time (see Teeling et al. [29]).

Bats are known to have emerged about 64 million years ago, in the Cretaceous-Tertiary boundary, once tropical climate prevailed throughout most of the globe (Fig. 1) [23, 29–31]. They originated in Laurasia (i.e., in the northern hemisphere) [29], when North America was still connected to Eurasia via the Bering Strait, and the African continent was already isolated from South America, which was indirectly connected to Australia by the Antarctic [32]. The earliest Chiropteran fossils were found in Europe, North America, Africa and Australia and date from the Eocene (49 million to 53 million years ago), when bats already appeared to be widespread [33]. Estimates from fossil anatomical analyses indicate that early bats were endowed with real flight capacity and displayed a type of echolocation [26, 34–36]. Thus, fossil records show that Eocene bats were fully developed with relation to the most important adaptive traits associated with Chiroptera, i.e. flight ability and echolocation [33]. The record of a bat fossil from the Eocene (*Onychonycteris finneyi*), which presented bone structures that could be interpreted as either a sign of non-echolocation or a primitive type of echolocation (see Simmons et al. [31], and Veselka et al. [37]) has given rise to some controversy on the origin of flight before echolocation in bats [31]. If flight evolved first in bats, then it could be possible that the first bats were diurnal, as it would be difficult to fly at night with no echolocating ability [31].

Flight capability is proposed to have influenced life history characteristics that led bats to be an exception among mammals. First, to fly efficiently, bats could not afford to have larger body sizes; thus, adaptive selection reduced body mass and favoured aerodynamics. While birds hold traits that contribute to flight efficiency, such as feathers and pneumatic bones, bats have membranes between fingers and legs and reduced ulnas [38]. Wing area does not proportionally increase with body mass in flying animals, so the maximum possible weight for bats is geometrically constrained. When body size increases, wing loading (i.e., body weight divided by wing area) also increases, consequently reducing flight maneuverability [39]. Despite being small-bodied—a characteristic usually associated with a fast life history strategy—bats have greater longevity, smaller offspring, and late sexual maturity than most similar-sized small mammals [39]. The increased vagility provided by their flight ability may have exposed Chiroptera to different selection pressures and possibly decreased their extrinsic mortality rates [40], which would consequently reduce the need for faster reproduction rates [39]. The lack of phenotypic trait-variation among bat species—uncommon in mammals—may imply that flight possession constrained drastic changes in bat life history as well as in bat morphology [39].

Flight innovation and echolocation capability were key factors increasing Chiroptera diversification and distribution around the globe [18]. Bats from the Eocene radiation preyed on insects and were nocturnal [33, 41]. As flying predators, bats might have had few competitors during the nights of the Eocene [42]. Additionally, bat nocturnal behavior could have evolved as a way to avoid daytime predators (e.g., hawks), which may have contributed to decrease their extrinsic mortality rates and to increase energy allocation for foraging [21]. This more efficient use of resources possibly contributed to the diversification of bat families during the Eocene. The origin of the major bat lineages coincides with an increase in the average annual temperature and with diversity peaks of plants and insects [29].

#### Diversification across phylogenetic scales

Diversification rates patterns vary greatly across bat lineages, higher than the ones found within other mammals [30]. Such variation in diversification rates generated a heterogeneous distribution in bat taxonomic diversity, with fewer families being rich in species (e.g. Vespertilionidae, Phyllostomidae and Molossidae) and several others are not, having less than 10 species (Fig. 1) [18, 30]. Key innovations within bats might have been responsible for different radiation events, which promoted changes in speciation rates across lineages [30]. Although changes in diversification rates may not be causally related with ecological innovations, many hypotheses have been proposed regarding diversification of certain bat families, such as Phyllostomidae and Pteropodidae [30].

The great diversity of Phyllostomidae has been attributed to the evolution of different dietary habits [19, 43–46]. Phyllostomids make the richest family of bats in the New World, (170 species; IUCN [47]) and drive bat richness patterns in the New World [18, 48]. Among all mammalian families, Phyllostomid bats show the greatest diversity of foraging strategies, accompanied by morphological, behavioral, and physiological adaptations to these strategies. Phyllostomidae is comprised by insectivorous, nectarivorous, frugivorous, carnivorous, omnivorous and hematophagous species [19, 49]. Among the phyllostomid subfamilies, the diversification rates of Stenodermatinae bats (predominantly frugivorous) followed the diversification of angiosperms [29, 30]. This may suggest that a mutualistic relationship in frugivory have had a key role in determining Phyllostomidae diversity [44].

Pteropodidae is a singular family: bats in family do not echolocate, are primarily frugivores or nectarivores, occur only in the Paleotropics, and are mostly diurnal [23, 42]. Phytophagy and diurnal habits of Pteropodidae are aligned with modifications in their teeth structure and visual orientation. Pteropodid bats also represent the most speciose family of the Old World and hold the largest known bats, commonly known as ‘flying-foxes’, some of them reaching 1 kg when adults [18, 50]. It is accepted that they have been distinct from other bats since the early Eocene [29]. This family experienced rapid radiations due to genetic drift in isolated populations, resulting in the current high taxonomic diversity, high morphological diversity in terms of body size, and high endemism [51].

The development of a complex echolocation system has been associated with the diversification of two bat families: Rhinolophidae and Hipposideridae. After Pteropodidae, these families are the most diverse in the Old World, with approximately 70 and 80 species, respectively [19]. The evolution of ‘high duty-cycle’ echolocation has allowed them (and one species of the Neotropical family Mormoopidae) to explore new niches detecting flying insects in enclosed environments, such as rainforests [27, 52, 53]. Sonar is more efficient when one pulse is not masked by echo from previous pulses. Bats deal with it by separating pulse and echo in time when using a ‘low-duty cycle’ system [22] or by separating pulse and echo in frequency when using high-duty cycle [27]. High-duty cycle echolocation provides Rhinolophidae and Hipposideridae with more efficient detection and capture of flying insects in areas with cluttered vegetation, which produces overlapping echoes, allowing greater specialization and selectivity in foraging [19, 54].

Although variation in morphology has been much discussed as a diversification driver in bats [19, 30, 43, 44], the family with the highest species richness (Vespertilionidae) is not highly morphological diverse. This observation has hindered work on phylogenetic relationships for this group [55, 56]. Vespertilionidae is the second largest family of mammals, with approximately 400 almost exclusively insectivorous widely distributed species [18]. While most bat families follow a negative latitudinal richness gradient (i.e., lower species richness at higher latitudes), Vespertilionidae species diversity increases towards north. Vespertilionid bats might have originated in North America [57], and they are the only bat family that reaches latitudes above 50°N [48, 56]. Vespertilionid lineages underwent rapid diversification (significantly faster than phyllostomids) [56], resulting in greater diversity within this clade. Conversely, phenotypical diversity did not significantly increase in vespertilionid bats as it did in phyllostomids. One probable reason might be because phenotypical diversification may be reduced among newly formed lineages, as reproductive isolation occurs rapidly in rapid diversification periods. In contrast to Phyllostomidae, the rapid diversification of Vespertilionidae in a temperate environment followed by a fast spread of species across almost all parts of the world may have reduced their need for morphological specialization [56]. Physiological and behavioral adaptations (e.g., hibernation and migration) as well as life history strategies (e.g., delayed fertilization and multiple offspring) may have allowed this family to populate new environments [58].

#### Macroecological diversity gradients

All species available for assembling local communities comprise a ‘regional pool’ (e.g., Carstensen et al. [59]) defined by a series of historically established abiotic filters determining species distribution in accordance with their requirements (e.g., Lessard et al. [13]). Due to the need for specific adaptations to abiotic conditions, environmental filters select species groups with similar traits within their regional species pools [60], which reflects the species sorting happening at large-scales to produce rules governing some of the macroecological diversity and distribution patterns [3].

For Chiroptera, the most important differentiation to be mentioned is the segregation in species pool composition between the Old and New Worlds. Notwithstanding bat increased dispersal capacity, the current arrangement of continents probably prevented biota exchange between these regions [61]. This pattern is also seen in other animals that are sensitive to low temperatures [62], which were unable to cross the Bering Strait a million years ago [32]. Emballonuridae, Molossidae and Vespertilionidae are the only bat families that are shared between the Old and New Worlds. Emballonuridae is confined to the southern hemisphere and is supposed to have reached the New World around 30 million years ago through a single dispersion event through the Atlantic [29]. Vespertilionidae and Molossidae are widely distributed over the northern hemisphere and may have used the Bering Strait as passage between the New World and the Old World [61, 63].

Chiroptera species diversity strongly varies with latitude, driving the global diversity gradient of mammalian species [20, 48, 64–68]. Bat species richness is higher in the tropics than in temperate regions, also exhibiting large variation in species composition [69]. As per above, replacement of tropical families (e.g, Phyllostomidae and Pteropodidae) by vespertilionid bats occurs towards higher latitudes, being this family the most dominating in temperate regions [48, 56]. Living at low temperatures costs energy [66, 70], and only few lineages overcame these energy constraints and developed cold tolerance to change their ranges towards temperate regions (e.g., Wiens and Donoghue [71]). Accordingly, Presley et al. [72] observed that while species distribution of rodents and birds along an altitudinal gradient varied with vegetation types, cold tolerance was more important to allow bats to be distributed in cold environments.

The ‘tropical niche conservatism’ hypothesis is a recognized biogeographical and evolutionary hypothesis [71] that could explain the latitudinal gradient of species richness for Chiroptera [20, 73] (but see Pereira and Palmeirim [74] and Arita et al. [57]). Tropical niche conservatism argues that several taxa were originated in tropical conditions because climate remained suitable for diversification over a large portion of Earth’s surface during most of the Tertiary period. These taxa would then be more diverse in the tropics because they have had more time to speciate. Additionally, niche conservatism contributed in a way that only a minority of bats within these lineages evolved adaptations that would allow expansion to colder and more arid climates of extratropical regions [71]. Buckley et al. [20] observed that the chiropteran richness latitudinal gradient follows tropical niche conservatism predictions (i.e., the gradient is stronger for more basal groups than for more derived groups). Since Chiroptera shared a common ancestor during a warm climate period of the early Tertiary [28], it is likely that these latitudinal patterns are governed by historical processes related to ecological zones of species origin, as proposed by the tropical niche conservatism hypothesis [71, 75].

The latitudinal chiropteran richness gradient seems to differ among continents. While species richness symmetrically increases from temperate to tropical zones in the Neotropical and Indo-Malayan regions (peaking in 120 and 100 species, respectively) [76], the Afrotropical region does not follow this symmetrical pattern and does not present high numbers of species coexistence [67, 76]. The unique bat species richness pattern of the African continent may have been particularly caused by the scarce existence of tropical forests, when compared to other continents. African bat diversity is positively correlated with the proximity to streams and lakes, and amplified by variation in topography [67]. Moreover, Africa has also been the continent with the largest rate of tropical forest area reduction since the Eocene (i.e., period of diversification of bats; Kissling et al. [77]), which may have contributed to species extinctions. Hitherto, the African continent has a large desert area that possibly limited the exchange of lineages across the evolutionary time (in relation to the adjacent temperate region)—a limitation that is less abrupt in other continents [78].

Since there is a significant difference between species pools of tropical and temperate regions, it seems that continental variation in bat species richness might be, in most part, determined by climatic variables rather than by factors related to phytogeographical zones [16, 79]. Compared to other mammals, bat geographical distributions have broader extensions [76]. Therefore, bat regional pools may not be predominantly limited to biomes [69, 80]. However, recent studies have shown that bat species previously believed to have broad ranges are actually made of a complex of small-ranged cryptic species, also supporting the reduced trait-variation within some chiropteran groups [81–83]. Despite their vagility, only few bat species (less than 3%) possess migratory behavior (i.e., seasonal movement of more than 50 km), with less than 0.016% of bat species being capable of migrating farther than 1000 km [58]. Most migratory bats belong to Vespertilionidae and migrate to survive seasonal temperature variation in temperate regions—despite hibernation being a more common response to seasonal change, even among migratory species [58].

In general, bat biogeographical diversity patterns follow a tropical gradient of species richness, with the exception of the family Vespertilionidae, which follows an inverse latitudinal diversity gradient, because of its distinct origin and adaptation to cold environments. As the only flying mammals, bats exhibit low morphological variation, but still present distinct foraging strategies and behaviour variation.

### Landscape scale: effects of habitat loss and fragmentation on bats

The landscape-scale concerns areas sufficiently large to allow the detection of environmental heterogeneity across the space [84]. The heterogeneous mosaic that represents a landscape consists of interactive units, which are types of landscape components [85]. Each species perceives the landscape mosaic according to its individual ecological characteristics, in a way that landscape extension and resolution will depend on the scientific question being addressed [86]. From this perspective, most studies at this scale aim at understanding how landscape composition (i.e. habitat loss) and configuration (i.e. habitat fragmentation per se) affect bat diversity. Most studies focus on the Neotropical region and on the family Phyllostomidae, creating a bias in the knowledge on the effects of environment on bats at the landscape scale towards this region and this group [87]. Additionally, many studies compare bat assemblages among certain landscape components, such as forests, farmlands, agroforestry systems and urban areas on bats (e.g. Faria et al. [88], Monadjem and Reside [89]). Studies interested in understanding the effects of patch size and isolation on bats, compare bat diversity in fragments or islands (e.g. Meyer and Kalko [90]). Other studies are focused in understanding the effects of variables measured at the landscape scale on bats, such as the percentage of forest cover, number of forest patches, or edge density (e.g. Estrada-Villegas et al. [91], Rodríguez San-Pedro and Simonetti [92]). The results of such studies indicate that landscape composition and configuration effects on bat assemblages and populations (i.e., species richness, composition, abundance) is ensemble- and species-specific in both tropical [87] and temperate zones [93, 94].

The adequate landscape size depends of the mobility of a species, which should always be addressed by the research question. Studies on bats use a high variability of landscape sizes, from smaller (i.e. circular landscapes of 250–1.5 km radius; e.g., Mendenhall et al. [95], Chambers et al. [96], Rocha et al. [97]) to larger landscape sizes (1–8 km, e.g., Mendes et al. [98], Gorresen et al. [99]). In summary, the strength of bat responses to landscape structure seems to be species- or guild-specific and dependent on the diversity response metric (e.g., species richness, species abundance, species evenness).

The role of habitat fragmentation as a barrier to bat populations dispersal or movement also depends on the bat ensemble in question [100–102]. High mortality rates crossing the matrix, inability to cross the matrix, and edge sensitivity could be responsible for a negative relationship between bat diversity and habitat fragmentation. However, bats can be either positive or negatively related to edge density and fragmentation [103–105]. In general, species associated with sedentary foraging modes, such as gleaning insectivores are more susceptible to fragmentation [15, 106]. Species with adaptations to fly inside vegetation present reduced flight speed and efficiency, and are more sensitive to forest reduction [93]. Frugivores feeding on the canopy are commonly recorded moving tens of kilometers each night, which is in accord with the discontinuous distribution of their resources across the landscape [15, 102]. Differences among foraging strategies and space use also are related to distinct responses to urbanization [107–110]. It has been demonstrated that urbanization responses are species-specific (e.g., Jung and Kalko [107]). This has been attributed to the ability of certain bat species to use new features and roosts formed from anthropogenic intervention [104, 109].

Bat occurrence relationship with landscape structure and vegetation type, which may be a result of bat mobility that allows species to exploit resources in small and isolated habitats [90, 111–114]. In fact, it has been seen that bat high dispersal ability ends up mediating a source-sink dynamic and allows the presence of species even in sub-optimal habitats [115]. Moreover, Presley and Willig [116] showed that species extinct in an island after a devastating hurricane could quickly recolonize it, depending only on the distance to the source. Thus, in accordance with their dispersal capacity, bats might be able to select where along their occurrence area they achieve higher fitness [91, 107, 115, 117]. However, as we have discussed in the paragraphs above, probably it will vary in accordance to species ensembles, which dictates bat occupancy and abundance along the landscape [118].

Spatial variation in environmental characteristics has been considered irrelevant in influencing bat beta diversity in Mexico (a country with great environmental heterogeneity) (e.g., Rodríguez and Arita [119]). However, López-González et al. [120] supported the idea that species composition and richness across Mexico are closely related to variation in environmental composition. These results suggest that species vagility does not reduce the importance of habitat features in the geographical distribution of bats, but flight makes a great advantage in bat selection for appropriate habitats [120]. It is likely that due to the ability to disperse across long distances and to temporarily use sub-optimal habitats [115], bats can cross unfavorable areas and, subsequently, establish in areas according to habitat specificity. In this way, bat species may present regionally extensive ranges of occurrences, however with differential distribution across the landscape.

Meyer and Kalko [90] evaluated at the same time the relative contribution of patch and landscape-scale variables for explaining species richness and compositional patterns in Panamanian land-bridge islands. At the patch scale, isolation distance from the mainland was the strongest predictor and bat assemblages were strongly structured by differential movement and species colonization ability. This agrees with the findings of Kalko et al. [102], where forest interior-dependent species (i.e. sedentary foraging behavior) are unable to maintain stable populations on islands, showing that water matrix is an important limitation to their dispersion. On the other hand, species with higher dispersal capacity (e.g. large frugivores) were randomly distributed across the archipelago structure, and they were abundant on even distant islands. Cisneros et al. [121] also found that forest-dependent species (as gleaning animalivorous) were more associated with landscapes that have shorter inter-patch distances (i.e., more forested regions) than species with greater dispersal capacity (e.g., many frugivorous and nectarivores species), although patch area is important in maintaining genetic diversity [122]. High mobile species were associated with areas more affected by human intervention (i.e., presence of plantations that provided resources to those bat groups).

In this section, we show that bat responses to landscape structure may be idiosyncratic among species and landscape metrics. Despite this, forest amount is often positively related to bat diversity and distribution. Bat vagility variation has also been considered a determinant in the relationship between species occurrence and landscape structure, with forest-dependent species negatively responding to habitat isolation and habitat loss.

### Metacommunity scale: species turnover and nestedness

Metacommunity studies evaluate the structure of a collection of communities at different sites often by evaluating patterns of turnover or nestedness among communities. Bat metacommunities are commonly found to have Clementsian structures, with groups of species replacing others based on the spatial or environmental gradient [115, 123, 124]. Nevertheless metacommunity structure also varies among guilds, with frugivores on Mexico having a quasi-nested metacommunity structure, nectarivores presenting a Gleasonian structure and insectivorous having a random metacommunity structure [125]. Nested metacommunities have small-ranged species distributions within broader-ranged species distribution, while Gleasonian metacommunities present species random turnover among communities.

An important factor affecting bat metacommunities is the spatiotemporal variation in resource availability [90, 103, 126, 127]. Determinants of metacommunity structure vary in accordance with the season, metacommunities may be structured by distance among patches during dry seasons and by the forest edge density during rainy seasons [90, 126]. Cisneros et al. [126] demonstrated that dynamics among habitats were primarily related to bat guilds, which determined whether species would disperse in the landscape. Since reproductive activities impose constraints on energy and increase nutritional demands, foraging behavior and home range size may differ seasonally [103]. Seasonal variation in metacommunity structure has been shown to differ across foraging guilds. Cisneros et al. [126] found that most bat species are randomly distributed during the rainy season, when resources are more abundant, while nectarivores and frugivores expanded their geographical distribution and animalivorous presented a checkboard pattern of distribution during the dry season.

In this section, we show that although the metacommunity scale of bat diversity has been studied, species distribution patterns do not consistently coincide because of both clade- and guild-specific responses, the influence of environmental gradients and the spatiotemporal variation in resource availability to bats.

### Local scale: habitat selection and interspecific interactions

**Fig. 2:**
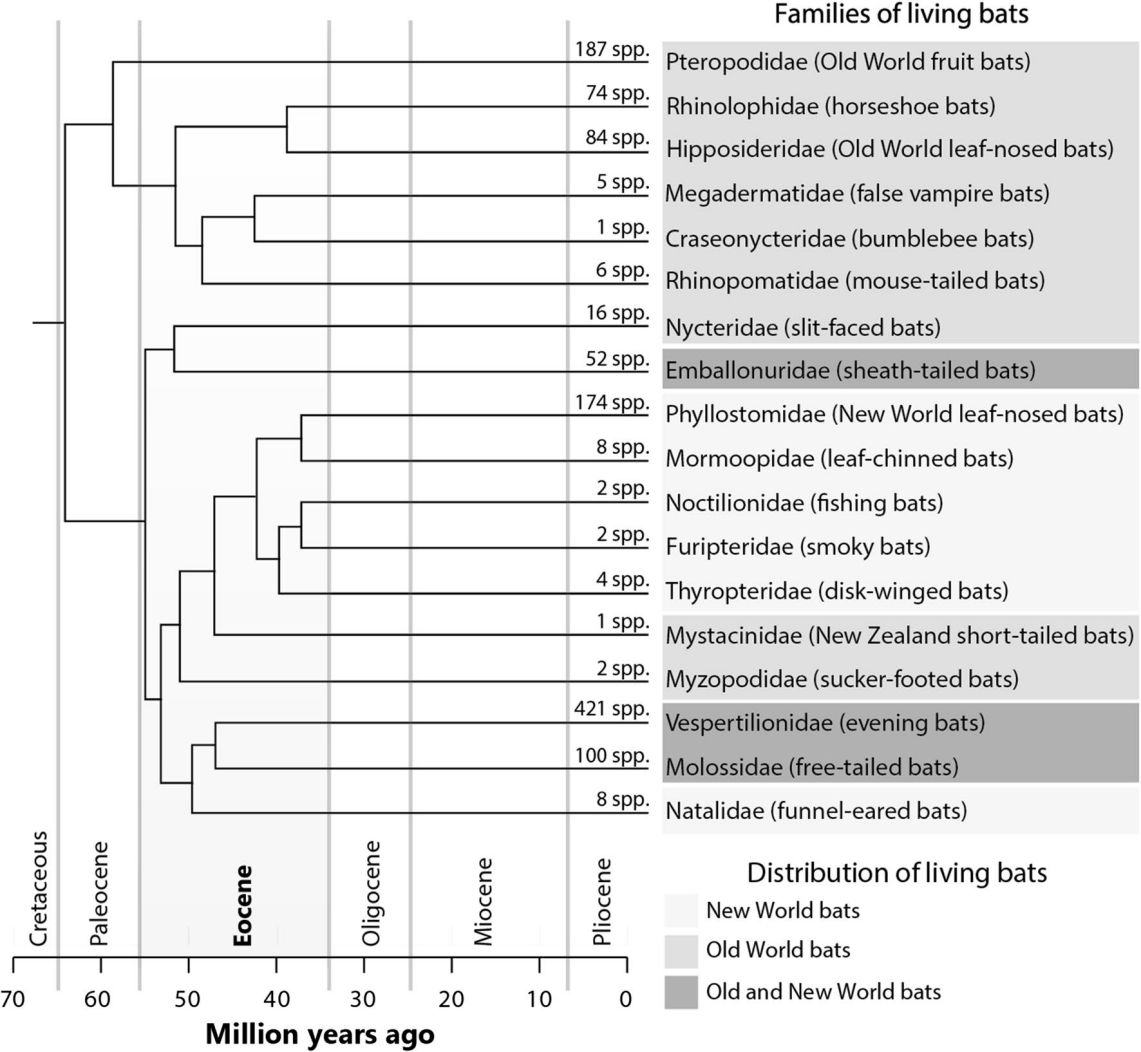
Temporal patterns of bat diversification, which occurred more intensively during the Eocene. Family and common names as well as number of species in each family are associated with each tip of the phylogenetic hypothesis tree. Gray shades represent the current geographical distribution of extant bats (see legend in right-bottom portion) (Adapted from Simmons [23] and Teeling et al. [29])

Bats exhibit scale-specific responses to human-driven modifications, where small-scale (i.e. local environment), medium-scale (i.e. landscape structure), and large-scale modifications (e.g. climate change) may simultaneously affect bat populations and assemblages [91, 97, 114, 118]. Local habitat characteristics define obstacles to flight and, understanding how different bat species perceive and deal with these obstacles is important to predict how bats use the landscape [128]. Morphological adaptations and echolocation type determine how bats use airspace according to the amount of obstacles or clutter in it: (1) open-space, by flying far from ground obstacles; (2) edge-space, using borders of vegetation patches and clearings, and; (3) narrow-space, flying inside the vegetation (Fig. 2) [15]. Bats foraging in open habitats do not need to deal with incoming echoes from the vegetation but instead they need to make detections over long distances. On the other hand, bats that use edge and interior vegetation need to receive location information of obstacles and even prevent these pulses to mask echoes that return from targets [15, 22]. High cluttered habitats decrease efficiency of prey detection because of an overlapping effect of the echolocation response. Some bat species deal with this issue by using high duty-cycle echolocation [27]. Hence, species foraging on this type of habitat usually use other senses to locate resources, such as olfaction (e.g., to find flowers and fruits) and hearing (e.g., to detect amphibian calls) [22]. Likewise, wing morphology differs with respect to the ability to perform maneuvers in an environment with obstacles. Wings from bats that stay at open-spaces tend to be narrower and longer, providing faster and energetically efficient long distance flights, while narrow-space bats have shorter and wider wings, which provide them with increased manoeuvrability [15, 22]. A remarkable distinction lies in Mystacinidae, which is the only bat family that developed morphological innovations for the use of the forest floor, instead of flying—like all other bats [129].

Differences in airspace use relate to resources that species can use, as well as, to their foraging strategy. Only species adapted to narrow spaces are able to forage within the understory, but most of species can exploit resources below the canopy [130]. Even among aerial insectivore bats, major differences have been seen in wing morphology, suggesting different foraging strategies [15, 93]. Similarly, among frugivorous species, there are those that forage in the canopy and those that feed around shrub vegetation. Therefore, even for species with similar diets, resource segregation might exist due to flight and echolocation adaptations. Characteristics of habitat use (i.e., wing morphology, echolocation type and foraging strategy) have been used to define guilds in Chiroptera [22]. The adaptations that dictate bat airspace use also influences individual home range [15]. The area used by bats is mainly influenced by the resources they are looking for, which is firstly determined by flight adaptations [102, 106, 131, 132].

In addition to habitat structure, local-scale biotic processes result from species interactions (such as competition and predation) and determine biodiversity patterns [1]. We have seen that Chiroptera presents a wide variety of foraging strategies, dietary traits, differential flight abilities and, consequently, habitat use. Thus, the definition of which species interact is directly linked to the knowledge on species behavior and morphology [15]. Based on foraging mode and habitat type, seven to ten bat guilds can be defined (see Fauth et al. [12], Denzinger and Schnitzler [22], and Kalko [15]). This variety of foraging strategies provides an extensive range of possibilities for interspecific interaction. Carnivorous and insectivorous bats are known to have coevolved with their preys, such as the evolution of an accurate echolocation system for prey detection and the evolution of hearing ultra-sound in moths [133, 134]. Mutualism between bats and plants has been associated with both bat and plant morphologies and geographical distribution, allowing bats to play relevant roles on seed dispersal and flower pollination [135, 136].

Based on the principle of limiting similarity between species co-occurring in a resource-constrained environment [137], competition has been one of the most tested local communities structuring process (e.g. Moreno et al. [138], Villalobos and Arita [14]). When compared to other mammalian orders, Chiroptera has a high degree of local species coexistence (up to 110 species in sympatry in the Neotropical region) [19]. This copious local diversity [i.e., alpha diversity (α)] has been attributed to niche partitioning owing to ecological diversity [19]. A decline in the average free niche space through packing (i.e., the tendency of species that coexist to fill possible ‘spaces’ along the niche dimensions) has been seen for different ensembles (sensu Fauth et al. [12]) in distinct continents [15, 139, 140].

Local communities appear to be a random sample of the regional species pool (including morphologically), suggesting that regional processes are more important in determining the composition and structure of bat ensembles rather than locally deterministic processes (such as competition) [14, 141, 142]. Although some of these studies have investigated the ecomorphological space and have performed analyses among guilds [14, 141], the spatial scale used may not have been sufficiently fine-scaled (e.g., Willig and Moulton [141]). These studies have included bat communities in large areas with high environmental heterogeneity and may not accurately reflect species group structure that co-occur in a particular habitat in a given time. When analyzing these patterns within ensembles (sensu Fauth et al. [12]) and at finer scales, limiting similarity between species (i.e., competition occurrence) has been found to be as an important process shaping community structure [138, 143, 144]. Large variation in home-range size may occur when in sympatry, even among species nearly morphologically identical and phylogenetically close [145]. Bat species may travel long distances to find resources or to avoid unsuitable places, but may remain in their current location if the habitats are suitable for their persistence (e.g., Cisneros et al. [121], Nicholls and Racey [145]). Vagility, thereby, may minimize temporal persistence of limiting similarity and hinder evidence of competition. Limiting similarity can only persist during periods when strong interspecific interactions are occurring. Species can easily return to previously occupied communities when resource availability increases [146]. The possibility to search for resources that flight provides may help species to avoid competition, even when similar.

Competitive processes that structure local communities can be inferred from analyses of other bat diversity aspects, such as the distribution of metacommunities (e.g., Cisneros et al. [121]). In the Peruvian Andes, for example, lower taxonomic diversity and higher phylogenetic and functional diversity were found at high altitudes, being associated with productivity declining potentially enhancing interspecific competition and resulting in competitive exclusion [121]. Such variation in phylogenetic and functional diversities, unrelated to variation in species richness, highlights the relevance of various processes in community structuring (e.g., environmental filtering, niche partitioning and competition) [8].

Predation is an interspecific interaction that contributes to determine community structure [1]. The high ability to avoid predators provided by flight in bats [40], does not prevent them to have predation as an important driver of their behavior and distribution, especially for tropical bats [147]. Bats are less active in full-moon nights and during twilight, probably to avoid predators, such as owls and falcons [148, 149]. Avoiding open areas is also a strategy to reduce exposure to predators [148]. However, predation by domestic animals seems to impact bat communities in human-altered environments [150]. Human pressure also threatens bat species due to hunt, mostly in the Paleotropics [151, 152].

Locally, bats select foraging and flight areas based in their vegetation structure. Wing morphology and echolocation mode is highly associated with the ability to fly in open or cluttered areas. Moreover, bats vagility with variation in the use of air space decreases chances of encounter among individuals of sympatric species, potentially the reduced rates of interspecific-competition found in some groups.

### Knowledge gaps

In this review, we have highlighted the importance of considering processes that occur hierarchically, from the global to a local community scale. Each process that occurs at a larger scale influences diversity patterns and will also determine how processes occur at smaller scales [1]. Thus, when we deal with different biota (e.g., Old and New World for bats; [61]), we likely deal with a different balance among processes across spatial scales. Nevertheless, there is large bias in bat research towards the New World, more specifically to the Neotropics. Filling this gap would improve the assessment of how historical differences in the formation of continents and barriers influence bat community structure. A lack of studies dealing with a mesoscale approach (i.e. landscape and metacommunity scales) represents a visible knowledge gap in the context of the scale gradient of diversity. Landscape ecology is a relatively new science [84] and most bat-specific studies dealing with the landscape scale are from the last few years. Studies that evaluate the importance of patch and landscape scale variables to explaining diversity patterns are still scarce, considering that they provide key information on conservation management of landscapes [87]. Moreover, a major difficulty is the correct definition of an appropriate scale, even though we considered the landscape scale hierarchically in a higher level than the metacommunity scale, some studies in the metacommunity scale are performed in broader areas (e.g., López-González [153], Presley and Willig [123]).

Comparing multiple measurements of bat diversity (i.e., taxonomic, phylogenetic and functional diversities) may help to understand the relative importance of different processes in structuring communities [121, 127]. This approach is more informative because it also takes into account the evolutionary history [154] and the characteristics that are directly affected by ecological processes (functional aspects) [155]. While other bat biodiversity aspects have been explored in studies that have addressed larger scales [156, 157], there is still a need to make progress at larger scales, as well as at scales that have had little attention (i.e., mesoscales and local scales).

## Conclusions

Among all hierarchical scales, we observed that the emergence of flight ability and the consequent major dispersal capability of bats influenced their diversification, life history, geographical distribution, and the intensity of local interspecific-interactions. Even though bats can disperse over long distances, limitations imposed by climate, such as temperature and precipitation, may have determined current diversity patterns at the continental level. High rates of species coexistence in Chiroptera is caused by increasing niche packing due to the large diversification of bats in the tropics. We also have seen that the efficient use of space and resources, which allows greater species coexistence, is also related to flight. Different flight, foraging and echolocation strategies combine to promote the differential use of air space, unique among mammals, and consequently increase the number of species supported by the environment. These different strategies are related to the home-range size, and therefore, to the way that the landscape is used by bats. There is a substantial trend in scientific research about bats to relate foraging/flight mode to how they will respond to environment and biotic conditions. This has been seen for all hierarchical scales approached in this review. The vastly majority of studies seems to confirm that there is in fact a relationship between the foraging and/or flight guild and ecological and evolutionary processes. Thus, from a scale perspective, it is important to understand the characteristics of the different ensembles of Chiroptera to understand the underlying ecological and evolutionary processes shaping bat diversity.

## Additional file

**Additional file 1.** Methods applied to perform this literature review on the main ecological and evolutionary processes underlying bat diversity patterns across hierarchical spatial and temporal scales.
