# Supplementary material for: A synthesis of ecological and evolutionary determinants of bat diversity across spatial scales
Source: BMC Ecol. 2018 Jun 11;18:18. doi: 10.1186/s12898-018-0174-z (PMC5996565; doi:10.1186/s12898-018-0174-z)
Supplement: Supplementary file 1 — Additional file 1. Methods applied to perform this literature review on the main ecological and evolutionary processes underlying bat diversity patterns across hierarchical spatial and temporal scales. [file 12898_2018_174_MOESM1_ESM.docx]

**Additional File 1 – Methods**

To review the main ecological and evolutionary processes underlying bat diversity patterns, we compiled scientific papers using the Web of Science database (version 5.14) on June 19^th^ 2015, and updated again on April 15^th^ 2018. Our search used the terms “Chiroptera OR bats OR bat” and “ecolog* OR evoluti*” and “diversity”, and included only studies published between 1945 and 2015. We intended to find most scientific manuscripts that include bat diversity as their response variable and that assessed the effects of ecological or evolutionary determinants on bat diversity. The above search terms were searched in 'Title', 'Abstract' and 'Keywords' fields. The terms “Chiroptera”, “bats” and “bat” include most common names used in scientific papers to represent bats. The terms “ecolog*” and “evoluti*” aimed to identify scientific papers that included “ecology”, “ecological”, “evolution”, “evolutionary” as well as all words containing such root forms. Subsequently, we selected scientific papers that addressed diversity determinant processes, and separated them across hierarchical scales by reading their Abstracts. All addressed processes and their corresponding scales were separated into: (1) global, (2) landscape; (3) metacommunity; and (4) local scales. Some studies were categorized across more than one scale. All cited scales above are in a hierarchical structure (*i.e.*, starting from the small habitat scale to the broad global scale), meaning that diversity outcomes of processes acting at larger scales can successively influence diversity structure on the smaller scales [1].

The initial search resulted in 1,287 studies, but only 126 were ultimately considered relevant and cited in this review. We also updated our search, adding 9 articles released from 2016 to 2018. This selection included scientific articles that were not found in our search but cited by one of found ones. We obtained data from (1) the hierarchical scale of the study, (2) which ecological or evolutionary processes were being associated with bat diversity, (3) which was the measurement of bat diversity, and (4) at which continent the study was developed. We did not aim to explore and define scale boundaries. Oppositely, we believe that scale boundaries vary among taxonomic groups, even among Chiroptera species.

**References**

1. Ricklefs RE, Schluter D. Species Diversity: Regional and Historical Influences. In: Ricklefs RE, Schluter D, editors. Species Divers. Ecol. Communities Hist. Geogr. Perspect. Chicago: Chicago University Press; 1993. p. 350–63.
